# Dietary diversity, food insecurity and the double burden of malnutrition among children, adolescents and adults in South Africa: Findings from a national survey

**DOI:** 10.3389/fpubh.2022.948090

**Published:** 2022-09-23

**Authors:** Abigail Harper, Jane Goudge, Esnat Chirwa, Alan Rothberg, Winnie Sambu, Sumaya Mall

**Affiliations:** ^1^Division of Epidemiology and Biostatistics, University of the Witwatersrand, Johannesburg, South Africa; ^2^Centre for Health Policy, University of the Witwatersrand, Johannesburg, South Africa; ^3^Gender and Health Research Division, The South African Medical Research Council, Johannesburg, South Africa; ^4^School of Therapeutic Sciences, University of the Witwatersrand, Johannesburg, South Africa; ^5^School of Economics, The University of Cape Town, Cape Town, South Africa

**Keywords:** dietary diversity, food security measurement, experiential indicators, stunting, obesity, national surveys, double burden of malnutrition, food expenditure

## Abstract

Childhood stunting remains a global public health problem. Many stunted children live in the same household as overweight or obese adults (the so-called double burden of malnutrition), evidence that quality as well as quantity of food is important. In recent years, food security measurement has shifted away from anthropometry (e.g., stunting) to experiential measures (e.g., self-reported hunger). However, given the continued problem of stunting, it is important that national surveys identify malnutrition.

**Objectives:**

To examine the associations between a variety of food security indicators, including dietary diversity, with adult, child (0–4 years) (5–9 years) and adolescent (10–17 years) anthropometry. To estimate the prevalence of double burden households.

**Methods:**

The study utilized cross-sectional data from the South African National Income Dynamics Survey NIDS (2008). We examined the associations between five food security indicators and anthropometry outcomes. The indicators were adult and child hunger in the household, self-reported household food sufficiency, food expenditure>60% of monthly expenditure and household dietary diversity. Multinomial and logistic regression models were employed to examine the associations with adult BMI categories and children's stunting and BMI.

**Results:**

The prevalence of stunting was 18.4% and the prevalence of wasting and overweight was 6.8 and 10.4%, respectively. Children <5 and adolescents with medium dietary diversity were significantly more likely to be stunted than children with high dietary diversity. Among children <5, child hunger and medium dietary diversity were significantly associated with wasting. None of the food security indicators were associated with stunting in children aged 5–9. Among stunted children, 70.2% lived with an overweight or obese adult. Among adults, increased dietary diversity increased the risk of overweight and obesity.

**Conclusion:**

Dietary diversity can be used as a proxy for poor nutritional status among children <5 years and adolescents but the relationship between dietary diversity and adult obesity is more complex. Given the double burden of malnutrition in many low- and middle-income countries, indicators of dietary quality remain important. These tools can be further refined to include an extra category for processed foods. Given the relative simplicity to collect this data, national surveys would be improved by its inclusion.

## Introduction

Stunting in children remains an urgent public health problem in low- and middle-income countries (LMIC). Research suggests that stunting is consistently associated with poorer cognitive function, schooling outcomes and reduced earning potential (1–3). However, stunting is a multifaceted problem associated with a variety of factors including chronic malnutrition, infectious diseases in early childhood and adverse birth and pregnancy outcomes like low birthweight and intrauterine growth restriction (3–6).

In many LMIC, there is an obesity epidemic occurring alongside high rates of stunting. This is primarily driven by fundamental changes in the food system including the ease and availability of cheap processed foods, reduced physical activity and the high cost of healthy varied diets (7, 8). Childhood stunting and adult overweight and obesity often occur in the same household, the so called double burden of malnutrition (DBM) (3, 7, 9–12).

In recent years, food security measurement has shifted away from the measurement of anthropometry to self-reported experiential measures. These measures, focused on individual perceptions of hunger and anxiety around access to food (13, 14), are premised on the concept that hunger and food insecurity are universal experiences that exist along a spectrum of severity (15). However, such measures do not capture the nutritional quality of food consumed. One indicator that has potential as an effective proxy for malnutrition are dietary diversity indicators. These have been found to be an effective indicator for overall dietary quality in some studies and are often associated with child anthropometry outcomes (16, 17).

A systematic review of household food insecurity and dietary diversity in relation to stunting in Sub-Saharan Africa (SSA) noted that two thirds of the included studies found that household food insecurity and low dietary diversity were linked to stunting (6). Another systematic review that examined dietary diversity and undernutrition across 32 demographic and health surveys in SSA found that children with adequate dietary diversity had a 12% lower likelihood of being stunted than those with inadequate dietary diversity (18). A study from Nigeria found that children's age, maternal age, and food expenditure were among the significant determinants of children's dietary diversity (19). Although the prevalence of food insecurity, low dietary diversity and stunting are subject to geographical variations, the linkages between them are often observed across regions. These findings suggest that both food insecurity and low dietary diversity are common in Sub-Saharan Africa and have adverse implications for growth and development, particularly among young children.

In South Africa, an estimated 27% of children under 5 years are stunted as a result of chronic malnutrition and a generally deficient growth environment (2, 3). The prevalence of stunting has remained virtually unchanged in the past two decades, despite a decrease in reported hunger in national statistics and one of the largest social protection systems in the world (2, 20, 21). National surveys in South Africa currently rely primarily on experiential measures of food security that measure hunger, skipping meals and running out of money to purchase food (the most severe forms of food insecurity). However, such measures may miss households that don't necessarily experience hunger but where child growth is faltering due to poor quality diets.

The findings from two recent national surveys suggest that food insecurity remains a serious problem in South Africa. Results from the 2017 general household survey (GHS) noted that about 13.4 million households had inadequate or severely inadequate access to food and about 1,6 million households experienced hunger (22). The NIDS coronavirus rapid mobile survey found that a lack of money to buy food remained high in 2020, due to the protracted nature of the COVID-19 pandemic and the subsequent economic and social impact (23, 24).

In this paper we estimate the prevalence of malnutrition among children, adolescents and adults and describe the proportion of double burden of malnutrition (DBM) households. Then, we examine whether different food security indicators are associated with adult BMI categories (normal weight, underweight, overweight or obese) and child height (normal height or stunted/severely stunted) and child BMI (normal weight, wasted/severely wasted and overweight/obese) and whether dietary diversity is an effective proxy for nutritional status.

## Methods

### The national income dynamics study

The South African National Income Dynamics Study (SA-NIDS) is a nationally representative panel survey of over 28,000 individuals in 7,300 households across South Africa. A stratified, two-stage cluster sample design was used in sampling the households to be included in the first wave (2008). In the first stage, 400 primary sampling units (PSUs) were selected from Stats SA's 2003 master sample of 3000 PSUs. This master sample was the sample used by Stats SA for its Labor Force Surveys and General Household Surveys between 2004 and 2007. The surveys were conducted on non-overlapping samples drawn within each PSU to ensure that households did not have to participate in both surveys (25). NIDS is a government funded survey to track inequality over time and examines several exposures (e.g., social capital, labor market participation, household composition and structure) in relation to poverty and inequality. Data on health outcomes, fertility and mortality were also collected. The survey is conducted by the Southern Africa Labor and Development Research Unit based at the University of Cape Town. Food security indicators were dropped in subsequent waves of the NIDS study and in this paper, we therefore use data from the baseline wave, conducted in 2008.

### Food security indicators

#### Household hunger

Data on adult and child hunger were collected separately using the following question: In the past year did an adult/child go hungry? never, seldom, sometimes, often, always. These are questions from the Household Food Insecurity Access Scale (HFIAS). We created a binary hunger indicator for adult and child hunger with households that reported never or seldom experiencing hunger scoring a 0 and households that reported sometimes, often or always experiencing hunger scoring a 1, using the same methodology as Statistics South Africa (26).

#### Food sufficiency

Respondents were also asked a question about household food consumption in relation to the household's needs in the past 12 months. Respondents reported if food consumption was less than adequate, just adequate or more than adequate for household needs. The data was converted into a binary indicator, with households who reported more than adequate or just adequate scored a 0 and households that reported less than adequate scored a 1.

#### Dietary diversity

We calculated household dietary diversity score (HDDS) from the NIDS data using the Food and Agriculture Organization (FAO) guidelines (27). The HDDS is comprised of 32 individual food types and 12 different food groups with a minimum score of 1 and a maximum score of 12. The 12 food groups are; (i) cereals and grain produces (ii) starchy roots and tubers (iii) Legumes (iv) vegetables (v) Fruits and nuts (vi) Sugars (vii) Meat and poultry (viii) Eggs (ix) Fish and shellfish (x) Milk and dairy products (xi) Oils and fats (xii) Miscellaneous (including beverages). Dietary diversity scores (DDS) was summed up by counting each of the 12-food groups, and classified as low (≤ 4), medium (5–8) and high (9–12) with high dietary diversity being the reference standard. There is no gold standard for dietary diversity cut-offs and we used these cut-offs based on a recent study that examined stunting and dietary diversity in South Africa (28). We also included dietary diversity as a continuous variable (ascending order from 1 to 12).

#### Food expenditure

Food expenditure was calculated by dividing the amount spent on food each month by total household expenditure. A cut-off of total monthly expenditure above 60% was used to define a household as food insecure, as recommended by the FAO (29, 30).

#### Child and adult anthropometry

Child anthropometry for children up to the age of 5 years was classified according to the WHO child growth standards, weight for height, BMI for age, and height-for-age (HAZ) scores. A HAZ score of −2SD of the mean is classified as stunted and a HAZ score of −3SD is classified as severely stunted. Child wasting and overweight/obesity was also classified according to the WHO growth standards with BMI for age below −2SD classified as wasted and BMI above 2SD classified as overweight (31). For children older than 5 years the WHO growth standards for school aged children and adolescents were used as a reference in the calculation of z-scores for height for age, BMI for age (32). Due to the low proportion of children who were severely stunted, we grouped stunted and severely stunted children together in the regression model. We also grouped wasted and severely wasted children together, and overweight and obese children together for the multinomial model. Child anthropometry data were calculated by the NIDS team (25). Children were grouped according to the following age categories: <5 years, 5–9 years and 10–17 years. People aged 18 and older were classified as adults and their BMI measurements were categorized according to the WHO growth standards and considered underweight (BMI <18.5), normal weight, (BMI 18.5–24.9) overweight (BMI 25–29.9) and obese (BMI >29.9) with normal weight used as the reference standard (33).

#### Data analysis

Logistic and multinomial regression models were used to examine child stunting and BMI status in relation to food security indicators. The explanatory variables were dietary diversity both as a continuous score and as a categorical indicator, food expenditure >60% of total monthly expenditure, child hunger in the past year and household food sufficiency in the past year. For the stunting model, the response variable was children's stunting status (normal height or stunted/severely stunted). For children's BMI the response variables were normal weight, wasted or overweight/obese. We examined each explanatory variable individually for both the stunting and BMI models (**Tables 4, 5**). Analyses were clustered at the household level on the assumption that children in the same household had similar access to food.

Multinomial regression models were used to examine adult BMI (underweight, normal weight, overweight and obese) in relation to food security indicators. The explanatory variables for both models were identical to the child variables except for hunger. The adult model used adult hunger as an explanatory variable in place of child hunger. For adult BMI, the response variable was adult BMI status. These results are presented in **Table 6**.

Food security indicators and outcome categories were generated from datasets with imputation values created by the NIDS data team (25). All analyses were conducted using Stata 15.1 (Stata Corporation, College Station, TX).

## Results

Insufficient food over the past 12 months was the most frequently reported food insecurity indicator (38.1%) followed by medium household dietary diversity scores (30.7 %). Low dietary diversity scores were the least common indicator (4.1%) followed by child hunger in the past 12 months (14.9%). These findings are presented in Table 1.

**Table 1:** Prevalence of household food insecurity by each item.

**Indicator (*n* households)**	**Percentage who reported food insecurity% (*n*) (95% CI)**
High dietary diversity (9–12)	65.2 (4 724) (64–66%)
Medium dietary diversity (5–8)	30.7 (2 219) (30–32%)
Low dietary diversity (1–4)	4.1 (297) (3–4%)
Food expenditure	17.3 (7 291) (16–18%)
Adult hunger	23 (7 266) (22–24%)
Child hunger	14.5 (5 359) (13%−15%)
Food Insufficiency	38.1 (7 291) (37–39%)

Height for age scores were available for 8 777 children across 3 831 household clusters. Children of normal height (*n* = 7 139) were the reference category. A total of 18.66% of children (*n* = 1 638) were classified as stunted as seen in Table 2. Children aged <5 years had the highest proportion of stunting (28.2%) followed by adolescents (17.9%). Children in the 5–9-year age category had the lowest prevalence of stunting (13.5%). These findings are presented in Table 2.

**Table 2:** Prevalence of childhood stunting by age category.

**Category**	**>5 years**	**5–9 yrs**	**10–17 yrs**	**Total**
	**% (*n*)**	**% (*n*)**	**% (*n*)**	**% (*n*)**
Normal height	73.8 (1 526)	86.5 (2 156)	82.1 (3 457)	81.3 (7 139)
Stunted/severely stunted	28.2 (543)	13.5 (338)	17.9 (757)	18.7 (1 638)
Total	100 (2 069)	100 (2 494)	100 (4 214)	100 (8 777)

BMI scores were available for 7 385 children across 3 559 household clusters. Children of normal weight (*n* = 6118) were the reference category. A total of 6.8% of children were classified as wasted or severely wasted (*n* = 500) and 10.4% of children were classified as overweight or obese (*n* = 767). The prevalence of wasting was highest among adolescents (7.7%) while the prevalence of overweight and obesity was highest among children aged <5 years (17.5%). These findings are presented in Table 3.

**Table 3:** Prevalence of childhood wasting and overweight by age category.

**Category**	** <5 years**	**5–9 yrs**	**10–17 yrs**	**Total)**
	**% (*n*)**	**% (*n*)**	**% (*n*)**	**% (*n*)**
Normal weight	77.2 (1 373)	85.7 (1 756)	84 (2 989)	82.8 (6 118)
Wasted/severely wasted	5.3 (95)	6.5 (133)	7.7 (272)	6.8 (500)
Overweight/obese	17.5 (311)	7.8 (160)	8.3 (296)	10.4 (767)
Total	100 (1 779)	100 (2 049)	100 (3 557)	100 (7 385)

For the full sample, each unit increase of dietary diversity offered a protective effect against stunting and reduced the risk of stunting by 5%. Children and adolescents children with medium dietary diversity were significantly more likely to be stunted than children with high dietary diversity (OR 1.35). When we stratified the children by age group, medium dietary diversity was significantly associated with stunting for children aged <5 years and adolescents. Low dietary diversity scores were associated with stunting among adolescents but not among other age groups. However, the prevalence of low dietary diversity was only 3.9% in this sample which likely contributed to the null finding. Medium dietary diversity and food expenditure >60% of monthly expenditure were associated with stunting among the adolescent group. None of the experiential indicators (child hunger and household food insufficiency) were associated with stunting for any age group in the sample. These findings are presented in Table 4.

**Table 4:** Logistic regression model of food security in relation to childhood stunting.

**Indicators**	**Odds ratio of being stunted (** ***p*** **-Value)**			
	**Bivariate regressions**			
	** <5 years**	**5–9 years**	**10–17 years**	**Full sample**
	***n* = 2,069**	***n* = 2,494**	***n* = 42,14**	***n* = 8,777**
Dietary diversity (continuous)	0.97 (0.195)	0.96 (0.153)	**0.91 (*****P*** **<** **0.000)**	**0.94 (*****P*** **<** **0.000)**
Medium dietary diversity (5–8)	**1.27 (0.028)**	1.12 (0.386)	**1.53 (*****P*** **<** **0.000)**	**1.35 (*****P*** **<** **0.000)**
Low dietary diversity 1–4	0.86 (0.628)	0.87 (0.667)	**1.88 (0.002)**	1.29 (0.115)
Food expenditure (>0.6)	1.26 (0.056)	1.09 (0.576)	**1.24 (*****P*** **<** **0.000)**	**1**.**24 (*****P*** **<** **0.000)**
Child hunger	0.97 (0.774)	0.99 (0.926)	0.97 (0.713)	0.97 (0.642)
Food insufficiency	1.08 (0.453)	0.89 (0.380)	1.14 (0.100)	1.07 (0.276)

Bold values indicate to highlight statistically significant results.

Medium dietary diversity and child hunger was associated with wasting in children <5. Child hunger represents the most severe form of food insecurity and households that reported children going hungry in the past year likely represent the most poor and deprived households. Each unit increase of dietary diversity decreased the risk of wasting in adolescents. Child hunger and food insufficiency decreased the risk of obesity among children in the 5–9-year age group and adolescents. These findings are presented in Table 5.

**Table 5:** Multinomial regression model of food security in relation to childhood wasting and overweight.

	** <5 years**	**5–9 years**	**10–19 years**	**Full sample**
**Indicators**	***n* =1,779**	***n* = 2,049**	***n* = 3,557**	***n* = 7 385**
**Relative risk ratio of being wasted or severely wasted (*****n*** **=** **500)**				
Dietary diversity (continuous)	0.93 (0.145)	0.96 (0.387)	**0.93 (0.017)**	**0.94 (0.008)**
Medium dietary diversity (5–8)	**1.76 (0.014)**	0.88 (0.541)	1.23 (0.160)	1.22 (0.086)
Low dietary diversity (1–4)	2.28 (0.071)	1.05 (0.802)	1.37 (0.389)	**1.88 (0.011)**
Food expenditure>0.6	1.51 (0.087)	0.81 (0.387)	0.81 (0.244)	0.92 (0.594)
Child hunger	**2.0 (0.003)**	1.11 (0.651)	0.97 (0.842)	1.17 (0.216)
Food insufficiency	1.0 (0.984)	1.16 (0.444)	0.95 (0.690)	1.01 (0.945)
**Relative risk ratio of being overweight or obese (n** **=767)**				
Dietary diversity (continuous)	0.98 (0.496)	1.06 (0.147)	1.05 (0.088)	1.03 (0.143)
Medium dietary diversity (5–8)	0.96 (0.782)	0.83(0.329)	0.87 (0.330)	0.89 (0.183)
Low dietary diversity (1–4)	1.35 (0.352)	0.84 (0.335)	0.93 (0.841)	1.04 (0.883)
Food expenditure>0.6	1.21 (0.189)	0.89 (0.607)	0.75 (0.098)	0.98 (0.841)
Child hunger	0.87 (0.392)	**0.50 (0.005)**	**0.56 (0.002)**	**0.66 (*****P*** **<** **0.000)**
Food insufficiency	0.92 (0.541)	**0.63 (0.011)**	**0.62 (0.001)**	**0.74 (*****P*** **<** **0.000)**

Bold values indicate to highlight statistically significant results.

Anthropometry measurements were available for 12 199 adults aged 18 and above across 6 483 household clusters. The prevalence of underweight, overweight and obesity was 7.6, 23.4 and 26.3%, (respectively). Adult hunger and household food insufficiency were the indicators most strongly associated with an increased risk of underweight (RR 1.25 and 1.34). Other food security indicators followed a similar pattern with an increased risk for underweight among adults and a decreased risk for overweight and obesity. However, each unit increase of dietary diversity increased the risk of overweight and obesity, but a reduction in dietary diversity was not associated with being underweight. These findings are presented in Table 6.

**Table 6:** Multinomial regression model of food security in relation to adult anthropometry.

**Indicators**	**Relative risk ratios (95% CI)** **Bivariate regressions**	***p-*Value**
**Relative risk ratio of being underweight (*****n*** **=** **927)**
Dietary diversity (continuous)	0.98 (0.96–1.01)	0.214
Medium dietary diversity (5–8)	1.09	0.321
Low dietary diversity (1–4)	0.94 (0.89–1.17)	0.730
Food expenditure>0.6	0.99 (0.85–1.19)	0.942
Adult hunger	**1.25 (1.10–1.45)**	**0.007**
Food insufficiency	**1.34 (1.10–1.43)**	***P*** **<** **0.000**
**Relative risk ratio of being overweight (*****n*** **=** **2,857)**
Dietary diversity (continuous)	**1.05 (1.03–1.07)**	**P <0.000**
Medium dietary diversity (5–8)	**0.86**	**0.003**
Low dietary diversity (1–4)	**0.70 (0.76–0.92)**	**0.004**
Food expenditure>0.6	0.90 (0.81–1.01)	0.087
Adult hunger	**0.67 (0.61–0.75)**	***P*** **<** **0.000**
Food insufficiency	**0.81 (0.74–0.90)**	***P*** **<** **0.000**
**Relative risk ratio of being obese (*****n*** **=** **3,219)**
Dietary diversity (continuous)	**1.11 (1.07–1.14)**	***P*** **<** **0.000**
Medium dietary diversity (5–8)	**0.69**	***P*** **<** **0.000**
Low dietary diversity (1–4)	**0.57 (0.52–0.65)**	***P*** **<** **0.000**
Food expenditure>0.6	**0.81 (0.71–0.91)**	**0.001**
Adult hunger	**0.71 (0.64–0.78)**	***P*** **<** **0.000**
Food insufficiency	**0.90 (0.80–0.96)**	**0.022**

The reference category was adults of normal weight N = 5,229. Bold values indicate to highlight statistically significant results.

There was a total of 3,720 households that had anthropometry measurements for both adults and children in the household and 850 (22.8%) of these households included stunted children as well as obese adults (Table 7). The DBM describes the coexistence of overnutrition (overweight and obesity) with undernutrition (stunting). In this sample, among households with stunted children, 70.2% of stunted children lived with overweight or obese adults. For ease of interpretation, we have grouped overweight and obese adults together as well as stunted and severely stunted children. When examining the double burden of malnutrition, households with one or more stunted child and one or more overweight or obese adults were classified as double burden households while households with stunted children and normal weight adults were classified as single burden households.

**Table 7:** Prevalence of the double burden of malnutrition by household.

**Category**	**Normal/underweight BMI**	**Overweight/obese adult**	**Total**
	**% (** ***n*** **)**	
Normal height child/ren	24% (602)	76% (1 908)	100 (2 510)
Stunted or severely stunted child/ren	29.8% (360)	**70.2% (850)**	100 (1 210)
Total	25.9% (962)	74.1 (2 758)	100 (3 720)

Bold values indicate to highlight statistically significant results.

## Discussion

Our results show that 18.43% of children are stunted, and that the double burden of malnutrition is evident in our sample with over 70% of stunted children living in the same household as an overweight or obese adult. Among children aged <5 years, children with medium dietary diversity are significantly more likely to be stunted than children with high dietary diversity. Among adolescents, medium dietary diversity, low dietary diversity and food expenditure are associated with stunting. Child hunger in the household and medium dietary diversity are significantly associated with wasting among children aged <5 years.

We did not find any of the food security indicators to be associated with stunting in children aged 5–9 years. There are several potential reasons for this. Children aged 5–9 had the lowest prevalence of stunting (13.5%) across age groups, with 28.2% of children <5 yrs and 18.6% of adolescents classified as stunted. The primary drivers of stunting among <5 yrs may be different (i.e., diarrhea and other infectious diseases or babies born small for gestational age) to those among older children. Moreover, stunting is a cumulative process and the consequence of chronic malnutrition and a deficient growth environment over time (3), hence the greater prevalence among adolescents. Thus, stunting in adolescence is a continuation from stunting in early childhood for most stunted adolescents. Adolescence is a critical period of development as 15–20% of total height is achieved during this phase. This may present the final opportunity to increase adult height but there is a lack of high-quality longitudinal evidence on whether catch up growth during adolescence is even possible (34). A South African cohort study found that found that <2% of children experienced late incident stunting between the ages of 2 and 5 (35). In other words, most of the linear growth deficit had already occurred by the age of 2 years. In addition, only a quarter of children who were stunted at age 2 experienced enough catch up growth to no longer be stunted by age 5 (35). Interventions to increase dietary diversity among vulnerable groups can still improve nutritional outcomes and wellbeing but this may not necessarily translate into a meaningful reduction in stunting (36).

Neither hunger nor food insufficiency were associated with stunting for any age group, highlighting the limitations of experiential indicators in relation to stunting. The South African General Household Survey (GHS) uses the Household Food Insecurity Access Scale (HFIAS), an experiential scale which classifies households into three separate categories of severity (food secure, moderately food insecure or severely food insecure) for monitoring food security at a population level (37, 38). The HFIAS was originally developed for food security surveys in the US population, where child stunting is very low and not considered a public health problem, unlike South Africa. Furthermore, responses to experiential scales like the HFIAS may vary dependent upon cultural and social contexts and this limits comparison of food insecurity prevalence across countries (16, 39). However, there is substantial evidence for the protective effect of household dietary diversity against childhood stunting and this has been observed in numerous studies from LMIC (6, 17, 40, 41). We found that medium dietary diversity was moderately associated with stunting among children <5 years and strongly associated with stunting among adolescents (OR 1.27 and 1.53). This suggests that children in the low to medium dietary diversity category are more likely to be malnourished and dietary diversity is an effective proxy for malnutrition.

While we did find that high proportion of food expenditure was associated with stunting among adolescents and reduced the risk of obesity among adults, expenditure data has several limitations as an indicator. These include that it is challenging to collect, and may be subject to recall bias and lacks generalizability across different regions and currency systems (16). However, food expenditure is associated with both children's linear growth and dietary diversity in a number of studies (19, 42). Furthermore, such data are routinely included in national surveys in many LMIC and so the association between longitudinal household food expenditure patterns, dietary diversity and children's linear growth could be the subject of more detailed research.

Although South Africa does not have public policies designed specifically to address childhood stunting, South Africa has an extensive Child Support Grant (CSG) program with over 12 million monthly disbursements to the caregivers of children aged 18 and under. The CSG is an unconditional cash transfer of R 400 (25 USD) per month to the primary caregiver. The CSG is intended to purchase food, school supplies and other essentials for low-income children. However, the CSG has not been effective in reducing the burden of stunting in South Africa. One potential reason for this is that the funds are insufficient to purchase even a basic food basket or that the funds are not used to purchase food (43, 44). However, some studies have found that when coupled with maternal education (grade 8 or higher) the CSG has a small but significant impact on increasing children's HAZ scores (45). These findings suggest that the CSG may be more effective over time if maternal education levels improve. A study from Mexico also found that maternal education mitigated the effects of child stunting and maternal overweight in a rural area (46).

However, the existing evidence suggests that even if they do experience catch up growth, children who were stunted at age 2 years perform almost as poorly in cognitive tests as children who remained stunted (35). This suggests that the first 2 years of life are critical for both linear growth and cognitive development and reinforces the need for interventions that can mitigate stunting in the first 1,000 days of life (35). Thus, improvements in household dietary diversity may reduce stunting during this critical period of development. However, dietary diversity needs to be consistently measured at the population level if policymakers are to identify vulnerable groups and develop effective interventions.

The double burden of malnutrition (DBM) is particularly common in LMIC countries like South Africa that have undergone a nutrition transition characterized by rapid changes in the food system and the availability of cheap and highly processed foods (7, 47). Of the stunted children in this study, over 70% lived in households with overweight or obese adults (Table 7). Many stunted children may not experience hunger but will still be malnourished by a nutrient poor diet that consists primarily of starchy staples. This “hidden hunger” may also extend to many of their overweight or obese parents. The double burden of malnutrition is also visible among stunted children who are also overweight or obese. Although this study found that *low* dietary diversity was associated with stunting, we also found an inverse relationship with adult BMI whereby *increased* dietary diversity was associated with being overweight or obese. However, increased dietary diversity did not increase the risk of overweight/obesity among children or adolescents. As the direction of the associations go in opposite directions for stunting and obesity, further research is also needed to elucidate the relationship between dietary diversity and anthropometry across the full income range.

A longitudinal analysis of NIDS data that examined changes in BMI found that higher household income per capita was associated with a higher rate of change in weight gain (48). Thus, an improvement in living standards and economic progress is also a driver of the obesity epidemic in South Africa. Cultural preferences around different body types, sedentary lifestyles as well as a lack of knowledge and education around healthy foods and nutrition also play a role (49–52). Discerning to what extent rising obesity rates are driven by higher income and broader choices of food, or food insecurity coping strategies such as increased consumption of cheap processed foods requires rigorous longitudinal research (7, 47).

### Limitations

While dietary diversity is a good proxy for dietary quality and micronutrient adequacy, it also has limitations, as most dietary diversity measures do not include a separate category for processed foods, an important risk factor for overweight and obesity (16, 17, 53). In addition, dietary diversity does not capture the quantities of the diverse foods consumed and there is a lack of formal cut-offs or theory that links a number of food groups consumed to nutrient adequacy or overall sufficient quantity of food (13). Currently, there is no gold standard dietary diversity measure and the most widely used scales vary from between 7 and 15 food different food groups (16).

## Conclusion

Stunting is a cumulative process and interventions to mitigate stunting at the beginning of the life course may be most effective for long term growth and developmental outcomes. Accurate monitoring of food and nutritional security at a population level is essential if LMIC hope to improve nutritional outcomes, particularly among vulnerable children. However, measures that are focused on hunger fail to capture important dimensions of dietary quality. Given the time and budget constraints of conducting large surveys, household dietary diversity data are relatively simple to collect and national surveys would be improved by their inclusion in addition to existing measures of food security.

## Data availability statement

Publicly available datasets were analyzed in this study. This data can be found at: http://www.nids.uct.ac.za/nids-data/data-access.

## Ethics statement

The studies involving human participants were reviewed and approved by the University of Cape Town (UCT) Commerce Faculty Ethics Committee. Approval for this secondary analysis of the NIDS data was approved by the Humanities Research Ethics Committee at the University of the Witwatersrand Research Ethics Committee (protocol number M1909101). Written informed consent to participate in this study was provided by the participants' legal guardian/next of kin.

## Author contributions

AH, SM, and AR were involved in conceptualizing the study design. AH performed the analyses while EC provided statistical oversight. WS assisted with data management and merging datasets across waves. AH, JG, and SM contributed to writing the article. All authors read and approved the final manuscript.

## Funding

AH was funded by the Sheiham Family Fellowship. The funding entails a 3-year fellowship to the Wits School of Public Health with a focus on the social determinants of health inequality in the South African context.

## Conflict of interest

The authors declare that the research was conducted in the absence of any commercial or financial relationships that could be construed as a potential conflict of interest.

## Publisher's note

All claims expressed in this article are solely those of the authors and do not necessarily represent those of their affiliated organizations, or those of the publisher, the editors and the reviewers. Any product that may be evaluated in this article, or claim that may be made by its manufacturer, is not guaranteed or endorsed by the publisher.

## References

[1] Dewey KG Begum K. Long-term consequences of stunting in early life. Matern Child Nutr. (2011) 7(Suppl. 3):5–18. 10.1111/j.1740-8709.2011.00349.x 21929633 PMC6860846

[2] May J Witten C Lake L. South African Child Gauge 2020. Cape Town: Children's Institute, University of Cape Town (2020).

[3] Leroy JL Frongillo EA. Perspective: what does stunting really mean? A critical review of the evidence. Adv Nutr. (2019) 10:196–204. 10.1093/advances/nmy101 30801614 PMC6416038

[4] Mosites E Dawson-Hahn E Walson J Rowhani-Rahbar A Neuhouser ML. Piecing together the stunting puzzle: a framework for attributable factors of child stunting. Paediatr Int Child Health. (2017) 37:158–65. 10.1080/20469047.2016.1230952 27680199

[5] Prendergast AJ Humphrey JH. The stunting syndrome in developing countries. Paediatr Int Child Health. (2014) 34:250–65. 10.1179/2046905514Y.0000000158 25310000 PMC4232245

[6] Gassara G Chen J. Household food insecurity, dietary diversity, and stunting in sub-saharan africa: a systematic review. Nutrients. (2021) 13:4401. 10.3390/nu13124401 34959953 PMC8707760

[7] Popkin BM Corvalan C Grummer-Strawn LM. Dynamics of the double burden of malnutrition and the changing nutrition reality. Lancet. (2020) 395:65–74. 10.1016/S0140-6736(19)32497-3 31852602 PMC7179702

[8] FAO IFAD UNICEF WFP WHO. The State of Food Security and Nutrition in the World 2021. Transforming Food Systems for Food Security, Improved Nutrition and Affordable Healthy Diets for All. Rome: FAO (2021). 10.4060/cb4474en

[9] Pourmotabbed A Moradi S Babaei A Ghavami A Mohammadi H Jalili C. Food insecurity and mental health: a systematic review and meta-analysis. Public Health Nutr. (2020) 23:1778–90. 10.1017/S136898001900435X 32174292 PMC10200655

[10] Leung CW Epel ES Willett WC Rimm EB Laraia BA. Household food insecurity is positively associated with depression among low-income supplemental nutrition assistance program participants and income-eligible nonparticipants. J Nutr. (2015) 145:622–7. 10.3945/jn.114.199414 25733480

[11] Laraia B Vinikoor-Imler LC Siega-Riz AM. Food insecurity during pregnancy leads to stress, disordered eating, and greater postpartum weight among overweight women. Obesity. (2015) 23:1303–11. 10.1002/oby.21075 25959858 PMC6563905

[12] FAO IFAD UNICEF WFP WHO. The State of Food Security and Nutrition in the World 2019. Safeguarding Against Economic Slowdowns and Downturns. Rome: FAO (2019).

[13] Cafiero C Melgar-Quiñonez HR Ballard TJ Kepple AW. Validity and reliability of food security measures. Ann N Y Acad Sci. (2014) 1331:230–48. 10.1111/nyas.12594 25407084

[14] Maxwell S,. Food Security: A Post-Modern Perspective. Brighton: Institute of Development Studies, University of Sussex (1994). Available online at: https://opendocs.ids.ac.uk/opendocs/handle/20.500.12413/3787

[15] Nord M. Introduction to Item Response Theory applied to Food Security Measurement: Basic Concepts, Parameters, Statistics. Technical Paper. Rome: FAO (2014). Available online at: http://www.fao.org/economic/ess/ess-fs/voices/en

[16] Headey D Ecker O. Rethinking the measurement of food security: from first principles to best practice. Food Secur. (2013) 5:327–43. 10.1007/s12571-013-0253-0

[17] Steyn NP Nel JH Nantel G Kennedy G Labadarios D. Food variety and dietary diversity scores in children: are they good indicators of dietary adequacy? Public Health Nutr. (2006) 9:644–50. 10.1079/PHN2005912 16923296

[18] Aboagye RG Seidu A Ahinkorah BO Arthur-holmes F Cadri A Dadzie LK. Dietary diversity and undernutrition in children aged 6-23 months in Sub-Saharan Africa. Nutrients. (2021) 13:3431. 10.3390/nu13103431 34684435 PMC8537414

[19] Otekunrin OA Otekunrin OA Ayinde IA Sanusi RA Onabanjo OO Ariyo O. Dietary diversity, environment and health - related factors of under - five children : evidence from cassava commercialization households in rural. Environ Sci Pollut Res. (2022) 29:19432–46. 10.1007/s11356-021-17221-y 34716896 PMC8556792

[20] Hunter W Patel L Sugiyama NB. How family and child cash transfers can empower women: comparative lessons from Brazil and South Africa. Glob Soc Policy. (2021) 21:258–77. 10.1177/1468018120981421

[21] Steyn NP Nel JH Drummond L Malczyk S Senekal M. Has food security and nutritional status improved in children 1– <10 years in two provinces of South Africa between 1999 (National Food Consumption Survey) and 2018 (provincial dietary intake study (PDIS)). Int J Environ Res Public Health. (2022) 19:1–21. 10.3390/ijerph19031038 35162059 PMC8834547

[22] Stats SA,. Towards Measuring Food Security in South Africa: An Examination of Hunger Food Inadequacy. Pretoria: Statistics South Africa, Private Bag X44, Pretoria 0001. (2019). Available online at: www.statssa.gov.za (accessed April 16, 2022).

[23] van der Berg S, Patel, L, Bridgman, G,. Hunger in South Africa: Results from Wave 4 of NIDS-CRAM Working Paper 11. (2021). Available online at: https://cramsurvey.org/wp-content/uploads/2021/05/11.-Van-der-Berg-S.-Patel-L.-_-Bridgman-G.-2021-Hunger-in-South-Africa-Results-from-Wave-4-of-NIDS-CRAM.pdf

[24] Spaull N Tomlinson M. Feeding a Family on $ 300 a Month. Business Live (2020).

[25] Leibbrandt M Woolard I De Villiers L. Methodology: Report on NIDS Wave 1. Technical Paper No.1. University of Cape Town (2009).

[26] Lehohla P. Food security and agriculture 2002 – 2011: In-depth analysis of the general household survey data Vol. IV. Pretoria, South AFrica: Statistics South Africa, Private Bag X44, Pretoria 0001. (2012). Available online at: http://www.statssa.gov.za/Publications2/Report-03-18-03/Report-03-18-032011.pdf (accessed May 4, 2022).

[27] Kennedy G Ballard T. Guidelines for Measuring Household and Individual Dietary Diversity. Rome: FAO (2010). p. 1–60.

[28] Modjadji P Molokwane D. Dietary diversity and nutritional status of preschool children in North West Province, South Africa : a cross sectional study. Children. (2020) 7:174. 10.3390/children7100174 33050271 PMC7600000

[29] Smith L Subandoro A. Measuring Food Security Using Household Expenditure Surveys. Food Security in Practice Technical Guide Series. Washington, DC: International Food Policy Research Institute (2007).

[30] Ryan J Leibbrandt M. Multidimensional Food Insecurity Measurement. Saldru Working Paper No 160. Cape Town: University of Cape Town (2015).

[31] WHO. WHO Child Growth Standards. Geneva: World Health Organization (2008).

[32] De Onis M Onyango AW Borghi E Siyam A Nishida C Siekmann J. Development of a WHO growth reference for school-aged children and adolescents. Bull World Health Organ. (2007) 85:660–7. 10.2471/BLT.07.043497 18026621 PMC2636412

[33] World Health Organization. Obesity: preventing and managing the global epidemic. Report of a WHO consultation. World Health Organ Tech Rep Ser. (2000) 894:1–253. 11234459

[34] Campisi SC Carducci B Söder O Bhutta ZA. The Intricate Relationship between Chronic Undernutrition, Impaired Linear Growth and Delayed Puberty?: Is 'catch-up' growth possible during adolescence? Office of Research - Innocenti Working Paper. Florence: UNICEF (2018).

[35] Casale D Desmond C Richter LM. Catch-up growth in height and cognitive function: why definitions matter. Econ Hum Biol. (2020) 37:100853. 10.1016/j.ehb.2020.100853 32036257

[36] USAID Nutrition Activities. Beyond Stunting Complementary Indicators for Monitoring Evaluating USAID Nutrition Activities (2021). Available online at: https://www.advancingnutrition.org/resources/beyond-stunting-complementary-indicators-monitoring-and-evaluating-usaid-nutrition (accessed June 15, 2022).

[37] Coates J Frongillo EA Rogers BL Webb P Wilde PE Houser R. What are we assessing when we measure food security? a compendium and review of current metrics. Adv Nutr. (2013) 4:481–505. 10.3945/an.113.004119 24038241 PMC3771133

[38] Cafiero C Viviani S Nord M. Food security measurement in a global context: the food insecurity experience scale. Meas J Int Meas Confed. (2018) 116:146–52. 10.1016/j.measurement.2017.10.065

[39] Leroy JL Ruel M Frongillo EA Harris J Ballard TJ. Measuring the food access dimension of food security: a critical review and mapping of indicators. Food Nutr Bull. (2015) 36:167–95. 10.1177/0379572115587274 26121701

[40] Kaibi FKM Steyn NP Ochola SA Plessis L Du. The relationship between agricultural biodiversity, dietary diversity, household food security, and stunting of children in rural Kenya. Food Sci Nutr. (2016) 5:243–54. 10.1002/fsn3.387 28265359 PMC5332258

[41] Chandrasekhar S Aguayo VM Krishna V Nair R. Household food insecurity and children's dietary diversity and nutrition in India. Evidence from the comprehensive nutrition survey in Maharashtra. Matern Child Nutr. (2017) 13:1–8. 10.1111/mcn.12447 29032621 PMC6866156

[42] Weingarten SE Dearden KA Crookston BT Penny ME Behrman JR Humphries DL. Are household expenditures on food groups associated with children's future heights in Ethiopia, India, Peru, and Vietnam? Int J Environ Res Public Health. (2020) 17:1–23. 10.3390/ijerph17134739 32630270 PMC7370180

[43] Devereux S Waidler J. Why does malnutrition persist in South Africa despite social grants? Food Security SA Working Paper Series No.001. DST-NRF Centre of Excellence in Food Security, South Africa. (2017).

[44] Khosa P Kaseke E. The utilisation of the child support grant by caregivers: the case of Ba-Phalaborwa municipality in Limpopo Province. Soc Work. (2014) 50:504–15.

[45] DSD SASSA UNICEF. The South African Child Support Grant Impact Assessment: Evidence Form a Survey of Children. (2012). Available online at: https://www.unicef.org/southafrica/media/1116/file/ZAF-South-African-child-support-grant-impact-assessment-2012.pdf (accessed May 4, 2022).

[46] Leroy JL Habicht J-P González de Cossío T Ruel MT. Maternal education mitigates the negative effects of higher income on the double burden of child stunting and maternal overweight in Rural Mexico. J Nutr. (2014) 144:765–70. 10.3945/jn.113.188474 24598879

[47] Popkin BM Gordon-Larsen P. The nutrition transition: worldwide obesity dynamics and their determinants. Int J Obes Relat Metab Disord. (2004) 28(Suppl. 3):S2–9. 10.1038/sj.ijo.0802804 15543214

[48] Cois A Day C. Obesity trends and risk factors in the South African adult population. BMC Obes. (2015) 2:1–10. 10.1186/s40608-015-0072-2 26617987 PMC4603579

[49] Venter F Walsh C Slabber M Bester C. Body size perception of African women (25-44 years) in Manguang. J Fam Ecol Consum Sci. (2009) 37:12–23. 10.4314/jfecs.v37i1.48942

[50] Mwaka1 NPSMLNNM. Affiliations: nutrition knowledge, attitudes and practices of primary school children in Tshwane. African J Prim Heal Care Fam Med. (2019). 10.4102/phcfm.v11i1.1846 PMC6556922 31038344

[51] Van Den Berg VL Okeyo AP Dannhauser A Nel M. Body weight, eating practices and nutritional knowledge amongst university nursing students, Eastern Cape, South Africa. African J Prim Heal Care Fam Med. (2012) 4:1–9. 10.4102/phcfm.v4i1.323

[52] Shisana O Labadarios D Rehle T Simbayi L Zuma K Dhansay A. The South African National Health and Nutrition Examination Survey, 2012 SANHANES-1. Cape Town: HSRC (2014). p. 423.

[53] Purushotham A Mittal N Ashwini BC Umesh KB von Cramon-Taubadel S Vollmer S. A quantile regression analysis of dietary diversity and health outcomes among children and women in the rural-urban interface of India. Food Policy. (2003) 107:6–8. 10.1016/j.foodpol.2021.102216

